# Spontaneous intracerebral hemorrhage associated with lenvatinib in advanced hepatocellular carcinoma without conventional risk factors: a case report literature review

**DOI:** 10.3389/fonc.2026.1875434

**Published:** 2026-06-17

**Authors:** Hyunmin Ji, Jeong-Ju Yoo, Sang Gyune Kim, Young Seok Kim

**Affiliations:** Division of Gastroenterology and Hepatology, Department of Internal Medicine, Soonchunhyang University Bucheon Hospital, Bucheon, Republic of Korea

**Keywords:** anti-angiogenic therapy, hepatocellular carcinoma, intracerebral hemorrhage, lenvatinib, VEGF inhibition

## Abstract

Lenvatinib is a multi-target tyrosine kinase inhibitor widely used in the treatment of advanced hepatocellular carcinoma (HCC). Its anti-angiogenic effects, however, may increase the risk of hemorrhagic events. Intracranial hemorrhage (ICH) during lenvatinib therapy is rare and remains poorly characterized. We report a case of spontaneous lobar ICH in a 54-year-old man with hepatitis B virus-related liver cirrhosis and advanced HCC with lung metastases. The patient was initially treated with atezolizumab-based therapy followed by lenvatinib due to disease progression. After seven months of treatment, he presented with a headache and visual disturbances. Brain magnetic resonance imaging (MRI) revealed a large lobar ICH in the right parieto–occipito–temporal region without evidence of metastatic tumor or vascular abnormalities. MRI spectroscopy did not support a malignant etiology. Although mild-to-moderate thrombocytopenia (platelet count 90, 000/µL) was observed, it was insufficient to fully explain the hemorrhage. The lesion initially improved but exacerbated subsequently, leading to discontinuation of lenvatinib. This case supports a possible association between lenvatinib therapy and spontaneous intracerebral hemorrhage even in the absence of hypertension or structural brain lesions, highlighting the need for constant vigilance in patients who present new neurological symptoms during treatment.

## Introduction

Lenvatinib is an orally administered, multi-targeted tyrosine kinase inhibitor that antagonizes vascular endothelial growth factor receptors (VEGFR) and fibroblast growth factor receptors (FGFR). It has been approved as a first-line systemic therapy for patients with unresectable hepatocellular carcinoma (HCC), based on non-inferior overall survival compared with sorafenib in the REFLECT trial (1, 2). While lenvatinib has demonstrated clinically meaningful efficacy, its anti-angiogenesis mechanism is associated with vascular adverse events, including hypertension and bleeding complications, which remain important considerations in clinical practice (3).

Intracranial hemorrhage (ICH) is a rare but potentially life-threatening complication in patients receiving anti-angiogenic therapies. Inhibition of VEGF signaling may impair endothelial integrity and predispose to vascular fragility and bleeding (4). Meta-analyses suggest that VEGFR-targeted tyrosine kinase inhibitors as a class are associated with an increased risk of hemorrhagic events (5). Although bleeding events related to lenvatinib have been reported in clinical trials and real-world cohorts, cases involving central nervous system hemorrhage during lenvatinib treatment remain limited (3).

Herein, we report a case of intracranial hemorrhage that occurred during lenvatinib therapy in a patient with advanced HCC. This report aims to delineate the clinical course, radiologic features, and potential mechanisms underlying this serious adverse event. This study was approved by the Institutional Review Board of Soonchunhyang University Bucheon Hospital (IRB No. SCHBC 2026-04-041; approval date: May-06-2026).

## Case report

A 54-year-old man first presented to the outpatient clinic on August 20, 2025, with a five-day history of headache and right periorbital pain.

The patient had a history of chronic hepatitis B virus infection but had not received prior antiviral treatment. He initially visited our hospital for evaluation of a lung mass and was subsequently diagnosed with HBV-related liver cirrhosis and advanced hepatocellular carcinoma (HCC) with multiple pulmonary metastases.

In March 2022, tenofovir disoproxil fumarate (TDF) treatment was initiated along with a combination systemic therapy of atezolizumab and bevacizumab. Bevacizumab was discontinued in January 2023 due to recurrent variceal bleeding, and atezolizumab monotherapy was continued thereafter. On January 13, 2025, follow-up imaging revealed a slight increase in the size of metastatic nodules in both lungs, indicating disease progression. The treatment was therefore switched to lenvatinib at a standard weight-based dose of 12 mg once daily (body weight: 66.5 kg), which was maintained for approximately seven months without dose reduction or interruption prior to the hemorrhagic event.

The patient had no other significant medical history or alcohol consumption. At initial presentation, vital signs were stable, with a blood pressure of 107/67 mmHg, a heart rate of 59 beats/min, a respiratory rate of 20 breaths/min, and a body temperature of 36.9 °C. No sign of hypertension was observed.

Neurological examination revealed left homonymous hemianopia. The patient also reported visual disturbance; however, no color vision deficit was identified. He was fully alert, and no other focal neurological deficits were observed, including deformities in motor, sensory, or cerebellar functions. Ophthalmologic evaluation showed normal visual acuity, pupillary reflexes, extraocular movements, slit-lamp examination, and fundoscopic findings, except for the visual field defect.

Laboratory findings were as follows: hemoglobin 13.8 g/dL, white blood cell count 3, 880/µL, platelet count 90, 000/µL, aspartate aminotransferase 43 IU/L, alanine aminotransferase 22 IU/L, sodium 134 mmol/L, potassium 4.3 mmol/L, chloride 103 mmol/L, total bilirubin 2.39 mg/dL, direct bilirubin 0.50 mg/dL, gamma-glutamyl transferase 50 U/L, alkaline phosphatase 129 U/L, lactate dehydrogenase 432 U/L, high-sensitivity C-reactive protein 0.12 mg/dL, and prothrombin time–international normalized ratio (PT-INR) 1.17.

**Figure 1:**
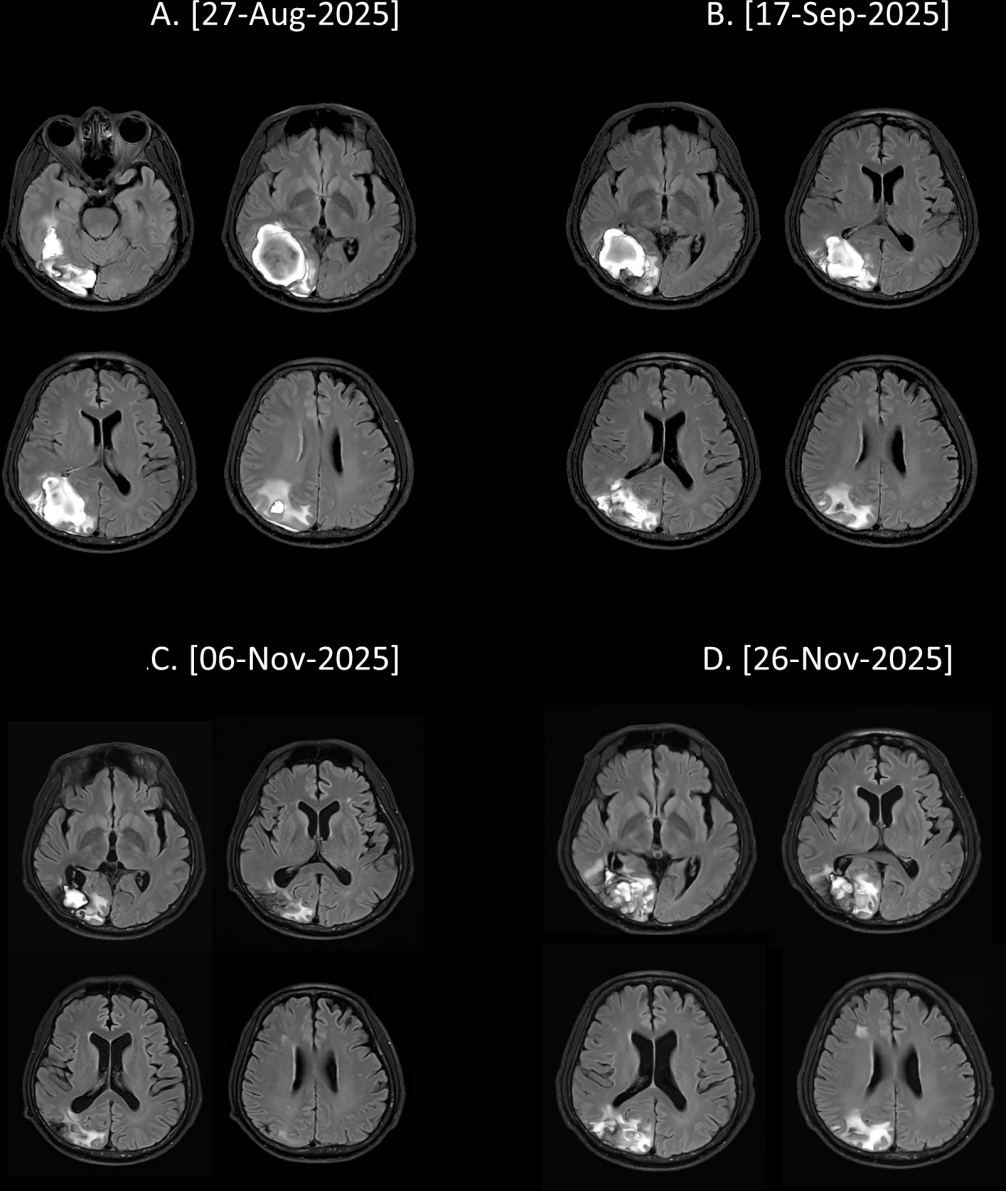
Serial brain MRI findings of intracerebral hemorrhage during lenvatinib therapy. **(A)** Contrast-enhanced brain MRI performed on August 27, 2025, demonstrated an approximately 6-cm acute-to-subacute lobar intracerebral hemorrhage involving the right parieto–occipito–temporal lobes, accompanied by surrounding vasogenic edema and irregular peripheral enhancement. **(B)** Follow-up brain MRI performed on September 17, 2025, showed a slight decrease in the size of the hemorrhagic lesion in the right temporo-occipital lobe, while the peripheral enhancing portion remained unchanged. Differential diagnoses included hemorrhagic metastasis and cavernous malformation with multistage hemorrhage. **(C)** Follow-up MRI obtained on November 6, 2025, demonstrated marked reduction in the size of the hemorrhagic lesion with decreased peripheral enhancement, reduced vasogenic edema, and resolving gliosis, suggestive of resolving multistage intracerebral hemorrhage. **(D)** Follow-up brain MRI performed on November 26, 2025, revealed interval aggravation of the previously identified intracerebral hemorrhage in the right temporo-occipital lobe during continued lenvatinib therapy.

Given persistent symptoms, brain magnetic resonance imaging (MRI) with contrast performed on August 27, 2025, demonstrated an approximately 6-cm acute-to-subacute lobar intracerebral hemorrhage (ICH) involving the right parieto–occipito–temporal lobes, with surrounding vasogenic edema and irregular peripheral enhancement (**Figure 1A**). Tumor bleeding from a solitary enhancing mass was initially suspected.

For further evaluation, the patient underwent close neurosurgical observation with serial neuroimaging follow-up. Additional imaging studies, including follow-up MRI and MRI spectroscopy, were performed in consultation with the neurosurgery team to further characterize the hemorrhagic lesion and exclude an underlying tumorous process. Follow-up brain MRI, including diffusion-weighted imaging, performed on September 17, 2025, showed a slight reduction in the size of the hemorrhagic component (approximately 5 cm) in the right temporo-occipital lobe. The peripheral enhancing portion remained unchanged, and no interval changes were found in other chronic hemorrhages in the bilateral frontal lobes and left cerebellum. The differential diagnosis included hemorrhagic metastasis versus cavernous malformation with multistage hemorrhage (**Figure 1B**).

Subsequent MRI spectroscopy performed on September 25, 2025, did not demonstrate metabolic findings suggestive of a malignant tumorous lesion, including the absence of a markedly elevated choline-to-N-acetylaspartate ratio around the lesion. Therefore, the lesion was considered more consistent with spontaneous intracerebral hemorrhage rather than metastatic tumor.

Follow-up imaging performed approximately two months later (November 6, 2025) demonstrated a marked decrease in the size of the hemorrhagic mass in the right temporo-occipital lobe, along with reduced peripheral enhancement, reduced surrounding vasogenic edema, and resolving gliosis (**Figure 1C**), findings consistent with resolving multistage intracerebral hemorrhage rather than progression of a tumorous lesion. Because serial imaging demonstrated interval improvement without progression of a definite enhancing mass, and because the causal relationship between lenvatinib and intracerebral hemorrhage remained uncertain at that time, lenvatinib therapy was temporarily continued with close imaging follow-up.

**Figure 2:**
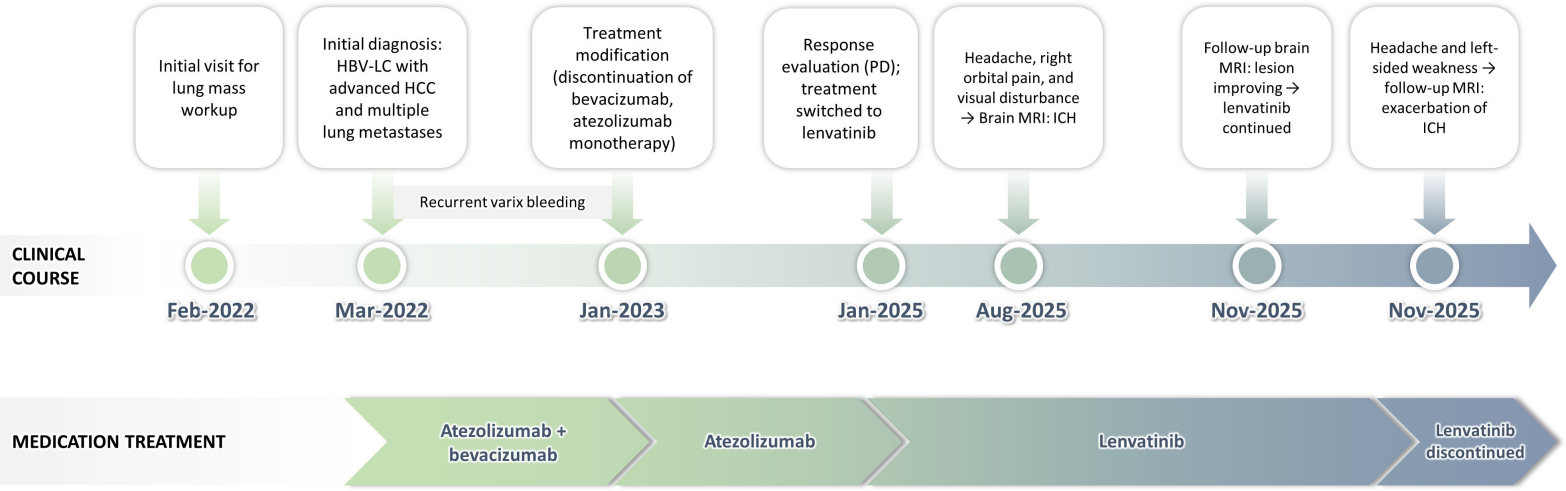
Timeline of systemic treatment, radiologic evolution, and clinical course. Timeline illustrating systemic anticancer treatment, onset and progression of intracerebral hemorrhage, serial neuroimaging findings, and interval aggravation during continued lenvatinib therapy.

Approximately two weeks later, the patient presented to the emergency department with an acute onset of headache, left-sided weakness, and nausea. Brain computed tomography (CT) and MRI with angiography performed on November 26, 2025, revealed re-exacerbation of the previously identified large ICH in the right temporo-occipital lobe (**Figure 1D**). Following admission for aggravated intracerebral hemorrhage, lenvatinib was discontinued. During hospitalization, the patient’s headache improved, and the previously noted left-sided weakness and nausea gradually resolved. The patient was subsequently discharged with a diagnosis of aggravated intracerebral hemorrhage. The overall clinical course, including the treatment timeline, onset of intracerebral hemorrhage, radiologic evolution, and re-exacerbation during continued therapy, is summarized in **Figure 2**.

## Discussion

This case describes a spontaneous intracerebral hemorrhage (ICH) that developed during lenvatinib treatment in a patient with advanced hepatocellular carcinoma (HCC). The patient had been switched to lenvatinib following disease progression after prior immune checkpoint inhibitor therapy and presented with headache and visual disturbance without a history of trauma or seizures. Neuroimaging confirmed the presence of hemorrhage; however, no definite structural causes, such as metastatic tumor, vascular malformation, or aneurysm, were identified.

Lenvatinib is a multi-target tyrosine kinase inhibitor that exerts its antitumor effects through inhibition of VEGFR 1–3, FGFR, and PDGFRα signaling pathways (1). Inhibition of VEGF signaling can impair endothelial integrity and pericyte support, resulting in structurally fragile vasculature prone to rupture (4). In addition, anti-angiogenic therapy may induce vascular remodeling and hemodynamic changes that further increase the risk of hemorrhage (4–6).

In this patient, no significant hypertension was observed during hospitalization, and the location of hemorrhage was inconsistent with the typical distribution of hypertensive ICH, making hypertension an unlikely primary cause of hemorrhage. Although the platelet count was 90, 000/µL, which may have contributed to increased bleeding tendency, it is unlikely to fully explain spontaneous lobar ICH. Instead, lenvatinib-associated endothelial injury and vascular fragility likely played a more substantial role. Cirrhosis-related thrombocytopenia, portal hypertension, and liver disease–associated coagulopathy may also have contributed to increased bleeding susceptibility. In addition, prior exposure to bevacizumab may have represented a potential predisposing factor for hemorrhagic complications, although the intracerebral hemorrhage developed after prolonged lenvatinib monotherapy.

The differential diagnosis included hemorrhagic brain metastasis, vascular malformation, aneurysm, cerebral amyloid angiopathy, cavernous malformation, and drug-related hemorrhage. At the initial presentation, the irregular peripheral enhancement observed on contrast-enhanced MRI raised concern for tumor-related hemorrhage. However, serial neuroimaging demonstrated gradual reduction of the hemorrhagic component along with decreased peripheral enhancement and surrounding edema, without progression of a definite enhancing mass lesion. In addition, MRI spectroscopy did not demonstrate metabolic findings suggestive of a malignant tumorous process, including the absence of a markedly elevated choline-to-N-acetylaspartate ratio. Brain MRI and MR angiography did not reveal a definite structural vascular lesion considered sufficient to explain the hemorrhage. Cerebral amyloid angiopathy was considered less likely given the patient’s relatively young age and the absence of characteristic imaging findings. Cavernous malformation was also considered less likely because serial neuroimaging did not demonstrate a definite underlying vascular lesion or progressive structural abnormality suggestive of cavernous malformation.

However, despite the apparent interval radiologic improvement, re-exacerbation of intracerebral hemorrhage subsequently developed during continued lenvatinib exposure. This clinical course suggests that temporary radiologic improvement may not necessarily indicate resolution of the underlying hemorrhagic risk during ongoing anti-angiogenic therapy. Although serial neuroimaging demonstrated temporary interval improvement of the hemorrhagic lesion, this likely reflected partial hematoma resorption rather than complete resolution of the underlying bleeding tendency. Persistent vascular fragility associated with VEGF pathway inhibition may have persisted despite apparent radiologic improvement. In addition, cirrhosis-related thrombocytopenia and liver disease–associated coagulopathy may have further contributed to ongoing bleeding susceptibility. Partial improvement of neurological symptoms following discontinuation of lenvatinib during hospitalization may be interpreted as being compatible with a possible dechallenge effect, although follow-up neuroimaging after discontinuation was not available. Based on the Naranjo Adverse Drug Reaction Probability Scale and the WHO–UMC causality assessment system, the association between lenvatinib exposure and intracerebral hemorrhage in this case was considered compatible with a possible to probable adverse drug reaction.

A review of previously reported cases of central nervous system hemorrhage associated with lenvatinib-containing regimens and other anti-angiogenic therapies (**Table 1**) demonstrates heterogeneous patterns in terms of hemorrhage type and timing. Although these reports involve different therapeutic combinations and clinical contexts, they collectively suggest a potential association between VEGF pathway inhibition and hemorrhagic complications (7–11). Notably, reports of spontaneous lobar ICH associated with lenvatinib monotherapy in the absence of structural lesions remain extremely limited, highlighting the distinctiveness of the present case.

**Table 1:** Reported cases of intracranial hemorrhage associated with lenvatinib-containing regimens and other anti-angiogenic therapies.

Case	First author (year)	Age/Sex	Primary malignancy	HTN	Regimen	Time to event	Hemorrhage subtype	Hemorrhage location	Imaging	Management	Outcome
1	Simão et al. (2023) (7)	60s/F	Endometrial cancer	NR	Lenvatinib + Pembrolizumab	NR	SAH	Bilateral frontal–parietal convexity	CT and MRI	Drug discontinuation, anticonvulsant	Alive
2	Iwasa et al. (2024) (8)	56/F	Renal cell carcinoma with brain metastasis	NR	Lenvatinib + Pembrolizumab + Stereotactic radiotherapy (SRT)	1 month	ICH (tumor-related)	Lobar	CT	Supportive care	Died
3	Ji (2026)	54/M	HCC	No	Lenvatinib monotherapy	7 months	ICH	Lobar (parieto-occipital)	CT and MRI	Drug discontinuation	Alive
4	Kim et al. (2025) (9)	70/M	HCC (alcoholic LC)	NR	Atezolizumab + Bevacizumab	After 2 cycles (2 days after cycle 2)	ICH with IVH	Right thalamus (with IVH)	CT and MRI	Supportive care	Died
5	Kim et al. (2025) (9)	56/F	HCC (HBV LC)	Yes (controlled)	Atezolizumab + Bevacizumab	After 2 cycles (1 day before cycle 3)	SAH	Anterior communicating artery (ACom) aneurysm	MRI	Coil embolization	Alive
6	Chao et al. (2023) (10)	76/M	Advanced HCC	NR	Atezolizumab + Bevacizumab	2 weeks	SDH with SAH	Convexity	CT and MRI	High-dose steroids (for irAE encephalitis)	Alive
7	Rinka et al. (2024) (11)	84/F	HCC (post-op recurrence)	NR	Atezolizumab + Bevacizumab (22 cycles; discontinued due to PD)	2 months after discontinuation (10 days after ITP treatment)	ICH (secondary to ITP)	NR	NR	Steroids + IVIG	Died

NR, not reported; SRT, stereotactic radiotherapy; IVH, intraventricular hemorrhage; SDH, subdural hemorrhage; SAH, subarachnoid hemorrhage; ICH, intracerebral hemorrhage; ITP, immune thrombocytopenia; irAE, immune-related adverse event.

^§^ Values approximated from graphical data.

With the increasing use of anti-angiogenic therapies, careful assessment of bleeding risk and close monitoring are essential (5, 6). In patients receiving lenvatinib, evaluation should include blood pressure, thrombocytopenia, and liver disease–related coagulopathy. Early neuroimaging should be considered in patients presenting with new neurological symptoms, even in the absence of hypertension.

In conclusion, although definitive causality cannot be established, this case supports a possible association between lenvatinib therapy and spontaneous lobar intracerebral hemorrhage, particularly after exclusion of more common structural and hypertensive causes. Clinicians should remain attentive for new neurological symptoms in patients receiving anti-angiogenic agents, as early recognition may help prevent serious complications.

## Data Availability

The original contributions presented in the study are included in the article/supplementary material. Further inquiries can be directed to the corresponding author.
